# Diet in Different Calcium Oxalate Kidney Stones

**DOI:** 10.3390/nu15112607

**Published:** 2023-06-02

**Authors:** Iris Coello, Pilar Sanchis, Enrique C. Pieras, Felix Grases

**Affiliations:** 1 Urology Department, Joan XXIII University Hospital, 43005 Tarragona, Spain; 2 Laboratory of Renal Lithiasis Research, University Institute of Health Science Research (IUNICS), Health Research Institute of the Balearic Islands (IdISBa), University of the Balearic Islands, 07122 Palma de Mallorca, Spain; 3 Urology Department, Son Espases University Hospital, 07120 Palma de Mallorca, Spain

**Keywords:** stone, food, nutrients

## Abstract

Diet can be a helpful tool to enhance the quality of urine and lower the likelihood and recurrence of kidney stones. This study set out to identify the foods and nutrients that are associated with each type of calcium oxalate kidney stone formation. A single-center, cross-sectional study was conducted. Between 2018 and 2021, a sample of 90 cases (13 cases with papillary COM, 27 with non-papillary COM, and 50 with COD kidney stones), as well as a control group of 50 people, were chosen. A food intake frequency questionnaire was completed by the study’s participants, and the results were compared between groups. Additionally, a comparison of the 24 h urine analysis between stone groups was made. Processed food and meat derivatives were linked to COM papillary calculi (OR = 1.051, *p* = 0.032 and OR = 1.013, *p* = 0.012, respectively). Consuming enough calcium may offer protection against non-papillary COM stones (OR = 0.997; *p* = 0.002). Similarly, dairy product consumption was linked to COD calculi (OR = 1.005, *p* = 0.001). In conclusion, a diet high in animal items may increase the risk of developing papillary COM stones. Consuming calcium may be preventive against non-papillary COM calculi, and dairy product consumption may be a risk factor for COD stones.

## 1. Introduction

Formation of kidney stones is a systemic disease that is becoming more prevalent and whose pathophysiology is complicated and involves many different factors, including age, race, other diseases, location, genetics, and diet. Renal calculi have increased in recent years and are expected to continue to rise. This evolution has been related to an increase in cardiovascular diseases as a consequence of lifestyle changes and food richer in salt and animal protein [1,2]. Given that pharmacological treatment requires taking medications for the entirety of a patient’s life with the associated financial cost and inconvenience, conservative management through changes in food and lifestyle are receiving more attention. By lowering urine supersaturation, raising crystallization inhibitor excretion, and lowering crystallization promoters, these modifications primarily aim to lower stone recurrence.

Changes in the anatomy of the urinary system along with variations in urine content lead to the formation of urinary stones, which is a complicated process. Calcium oxalate stones represent 70% of all types of nephrolithiasis and are subdivided in two groups: calcium oxalate monohydrate (COM) and calcium oxalate dihydrate (COD) stones. COM stones usually appear in context of an inhibitory crystallization factors deficit and are subdivided in two subtypes. On the one hand, papillary COM stones are formed from subepithelial hydroxyapatite calcification caused by urothelium damage that, when extruded in the renal papilla and in contact with urine, originate a nidus on which this type of calculi will develop. On the other hand, non-papillary COM stones are formed in renal cavities with low urodynamic efficacy. Finally, COD stones develop more frequently under conditions of high Ca urinary concentrations (hypercalciuria), normal or low citrate concentrations, and pH ≥ 6 [3]. It is important to consider that COD crystals are thermodynamically unstable, and they transform into COM crystals in contact with urine. For this reason, the presence of COM in COD calculi is due to this transformation, which in some cases can be complete, and they can be detected using electron microscopy [4].

Currently, there is a paucity of high-level evidence to confirm the effect of diet on kidney stone formation, and current results are controversial [5,6,7,8,9]. It could be due to difficulties in organizing and implementing a study assessing changes in diet and stone formation and recurrence. In addition, many different factors are involved in diet, and a multivariate analysis (MA) is essential for the study. Further, most studies have not taken into account types of kidney stones, or they simply refer to calcium oxalate stones in general and not to their subtypes.

Therefore, it is of special interest to study which diet factors, aimed at qualitatively modifying the urine, can help prevent the incidence of stone disease, given that it is an economic intervention that can reduce the impact on the quality of life of affected individuals and on public health resources.

## 2. Materials and Methods

An observational, case-control, cross-sectional, and unicentric study was carried out between 2018 and 2021. The case group (with kidney stones) was composed of 90 patients subdivided into papillary COM, non-papillary COM, and COD kidney stones (13, 27, and 50 cases, respectively), and the control group comprised 50 individuals without previous history of urolithiasis. Patients in preventive treatment for lithiasis or with anatomical alterations in the urinary tract were excluded. All patients included in this study provided informed consent, and this investigation was approved by the Ethics Committee of the Balearic Islands (CEI-IB) (nº IB 3714/18).

All cases included in the study completed a food frequency questionnaire (Predimed) [10], and the calculi obtained via surgery or spontaneous expulsion were analyzed. A 24 h urine analysis was obtained from each case. Moreover, a fasting 2 h urine collection was also obtained in the morning, to accurately determine the basal pH value of each individual. Both the 24 h urine analysis and food frequency questionnaire were completed between 1 and 3 months after expulsion or stone surgery. All controls completed the food frequency questionnaire.

Kidney stone groups considered were: papillary calcium oxalate monohydrate (COM), non-papillary COM, and calcium oxalate dihydrate (COD). To identify and classify these kidney calculi, it is necessary to establish the specific region of origin, the presence of different crystalline phases, the sequence of their development, and their relationship with renal morphoanatomy and urine composition. This information can only be obtained from a morphocompositional study of kidney calculi (combination of stereoscopic microscopy, scanning electron microscopy (SEM) + elemental microanalysis using EDS, and infrared spectroscopy) [3,4].

**Figure 1:**
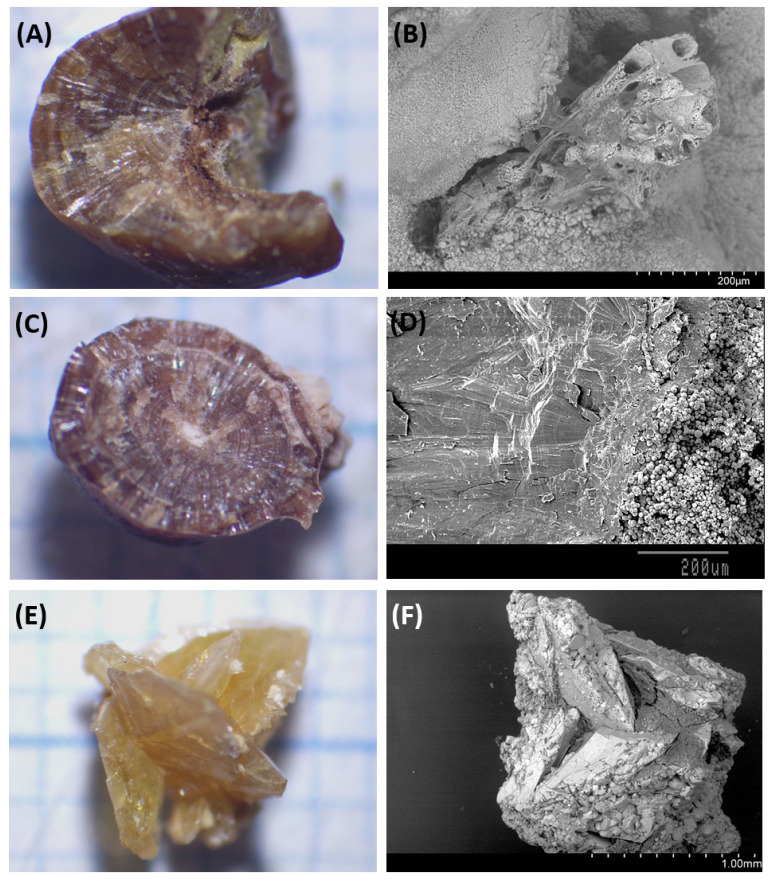
Typical examples of the studied calculi. COM papillary calculus: (**A**) General view of the section (**A**); SEM image of tubules in the concave zone of union to the papilla (**B**). COM non-papillary calculus: general view of the section (**C**); SEM image of hydroxyapatite localized in the center of the calculus (**D**). COD calculus: General view of the calculus (**E**); SEM image showing the presence of a small amount of hydroxyapatite and important transformation of COD to COM crystals (**F**).

The first step in studying COM calculi (papillary or detached) is to use a stereoscopic microscope to observe the outsides of the objects. To determine the underlying structures and locate the calculi’s cores, each calculus is divided into two halves along a plane that is as close as feasible to its geometric center. A typical papillary COM stone primarily comprises a radially striated convex outer layer and an excentrical core that is situated close to the concave area of the stone where it was connected to the papilla [3,4]. Microcomponents in the core can be found using scanning electron microscopy, and the concave exterior cavity can be used to confirm the calculus’s papillary origin. Thus, the existence of a point of attachment to the renal papillae is shown via the abundance of organic debris and tubular apical cells [4], as can be observed in Figure 1A,B.

A typical unattached (non-papillary) COM calculus basically consists of a symmetrically round stone with a central core surrounded by columnar COM crystals emerging from the core (containing the heterogeneous nucleants) and is marked by the absence of a site of stone attachment to the epithelium, as shown in Figure 1C,D [4].

Calcium oxalate dihydrate (COD) stones include stones that can contain small amounts of apatitic phosphates (<10%) and that can contain very high amounts of calcium oxalate monohydrate (COM), as a result of the transformation of COD crystals to COM crystals, due to urine-mediated processes. Using electron microscopy, it is possible to perfectly distinguish between the COM crystals formed directly and those that come from the transformation of COD crystals, as is shown in Figure 1E,F [3,4].

The food frequency questionnaire used was the Predimed questionnaire [10]. This questionnaire provides highly reproducible data regarding food and nutrient intake. It includes 137 foods, and frequencies of food consumption are reported on an incremental scale with nine levels (never or almost never, 1 to 3 times a month, once a week, 2 to 4 times a week, 5–6 times a week, once a day, 2–3 times a day, 4–6 times a day, and more than six times a day). Through the information regarding each food and its frequency of consumption, a nutritional value for each individual diet was obtained.

For quantitative variables with normal distribution, Student’s t-test for independent samples was used to compare the variables between two groups. For more than two groups, the two-tailed ANOVA test was used to determine the significance of the differences, and the Bonferroni test was used as a post hoc test to assess differences between various groups. For quantitative variables with non-normal distribution, non-parametric Kruskal–Wallis and U Mann–Whitney tests were used.

The relationship between the most important dietary factors associated with each calculi type was also studied using univariate logistic binary regression. In addition, in order to establish the most relevant dietary factors, multivariate logistic regression was used for each type of kidney stone after adjusting for confounding variables (age, sex, and BMI). In these models, only those dietary variables with a significant association for each type of calculi in the univariate analysis (*p* < 0.1) were included.

## 3. Results

A total of 90 individuals were included in this study. In Table 1, clinical differences between the control group and different kidney-stone-type groups are represented. Comparison of 24 h urine analysis between all stone groups is represented in Table 2.

### 3.1. Papillary COM Stones

This group included the most consumers of meat and derivatives (256 ± 139 g/day) and sausages (27 ± 35 g/day), and it showed a significantly higher consumption of dairy products, meat and derivatives, and sausages compared to the control group (*p* < 0.05). Additionally, this group showed a higher intake of protein, lipids, cholesterol, and trans fatty acids (TFAs) in comparison to the control group with a statistically significant difference (128 ± 38 g/day vs. 112 ± 84 g/day; 130 ± 54 g/day vs. 12 ± 113 g/day; 634 ± 214 mg/day vs. 513 ± 330 mg/day; 1.7 ± 0.8 g/day vs. 0.9 ± 0.9 g/day, respectively) (*p* < 0.05). Furthermore, MA showed that meat and derivatives, sausage, and TFA intake could be a risk factor for papillary COM stones (OR = 1.012, *p* = 0.012; OR = 1.051, *p* = 0.032; OR = 9.732, *p* = 0.004, respectively) (Table 3). Regarding micronutrients, the papillary COM stone group was highlighted as being the largest consumer of sodium. Furthermore, it showed significantly higher consumption compared to the control group (3635 ± 1605 mg/day vs. 2943 ± 2987 mg/day, *p* < 0.05).

Nevertheless, no statistically significant differences in pH, urinary urate, and citrate were observed between COM papillar, COM non-papillar, and COD groups. In addition, no differences in protein consumption between these three groups were observed.

### 3.2. Non-Papillary COM Stones

The group that showed the lowest calcium (Ca) intake was the non-papillary COM group (991 ± 448 mg/day). In addition, MA showed that Ca intake could be a protective factor for non-papillary COM stones (OR = 0.997; *p* = 0.002) (Table 4).

In both papillary and cavity COM calculi, urinary biochemical tests did not reveal remarkable alterations, as usual. The only alteration previously described is hyperoxaluria, but it was not observed in our data.

### 3.3. COD Stones

The COD stone group showed significantly higher dairy product intake than the control group (*p* < 0.05), and MA showed that this could be a risk factor for COD stones (Table 5). This group was the largest consumer of legumes of all groups (35 ± 48 g/day) and was the group with the highest intake of phytate (962.5 ± 898.9 mg/day). 

## 4. Discussion

### 4.1. Papillary COM Stones

The papillary COM group stood out as being the group that consumed the most meat products and derivatives (256 ± 139 g/day) and sausages (27 ± 35 g/day) of all groups. Additionally, compared to the control group, it showed a considerably larger consumption of meat and derivatives as well as sausages (*p* < 0.05). In relation to this food pattern, this group showed significantly higher consumption of meat-related macronutrients (proteins, lipids, cholesterol, and trans fatty acids). Further, MA showed that meat and derivatives, sausage, and trans fatty acid (TFA) intake could be a risk factor for papillary COM stones (OR = 1.012, *p* = 0.012; OR = 1.051, *p* = 0.032; OR = 9.732, *p* = 0.004, respectively). These data support the results of published studies to date that defend a relationship between meat intake and kidney stones. A recent meta-analysis, published by Bing-Biao et al. in 2020, which included 48 studies on diet and kidney stones, showed that a high intake of meat increased kidney stone risk significantly (RR: 1.24; 95% CI 1.12–1.39) [11]. Furthermore, a prospective cohort study carried out in the UK by Littlejohns et al., also published in 2020, noted an increased lithogenic risk with high meat intake [12]. 

On the other hand, high meat and derivatives and TFA consumption may imply high oxidative stress in renal tissue, which could play an important role in the genesis of papillary lesions characteristic of this type of stone [3].

Furthermore, the papillary COM group has demonstrated the highest sodium intake. Some studies have defended the relationship between a high sodium intake and increased urinary Ca excretion and an increased risk of kidney stones [11,12]. Our data may support these investigations; however, this group did not reveal hypercalciuria. It should not be forgotten that 24 h urine is a spot measurement and may not always be accurate. In addition, it must be remembered that the quantification of the use of table salt as well as hidden salt in processed foods make research of sodium in the diet highly complex. Once again, high sodium intake could be related to hypertension, which can also play a role in the origin of papillary lesions [13]. 

The COM papillary stone group showed the highest citrate urine excretion. This information is of interest because some research has linked COM calculi to a lack of crystallization inhibitors. This group did not consume a lot of fruits and vegetables, which are the primary sources of citrate; hence, the citraturia is surprising. Nevertheless, it should be noted that juices, a common habit and part of everyday consumption in our society that may contribute to citraturia among other uncontrollable factors, were not examined in this study. However, there were no statistically significant differences between papillary COM, non-papillary COM, and COD stones in terms of urinary citrate. Even so, citraturia would be interesting to examine when compared with the control group and not only between stone groups.

Regarding 24 h urine volume, the papillary COM stone group showed the lowest value, which could be justified by the low consumption of fruits and vegetables. However, no significant differences in urine volume were observed between groups in this study. Finally, creatinine, a substance produced by muscle activity or large meat intake, is more concentrated in this group (140 ± 63 mg/dL in 2 h urine and 113 ± 43 mg/dL in 24 h urine). It has been noted that the group with papillary COM lithiasis had the lowest volume of diuresis, which could account for the high levels of creatinine in urine together with a high meat intake.

### 4.2. Non-Papillary COM Stones

The group that showed the lowest Ca intake was the non-papillary COM group. In addition, MA showed that the intake of Ca could be a protective factor against non-papillary COM stones (OR = 0.997; *p* = 0.002). Several studies have argued that poor Ca intake, especially CaOx, is associated with a higher stone risk. Ca binds to oxalate in the intestinal lumen, preventing absorption of the latter, so that only free oxalate and Ca are absorbed. Therefore, a poor Ca intake can be reflected as an absorptive hyperoxaluria. It is noteworthy then, that this group, with the lowest intake of Ca even below the recommended amount (1–1.2 g/day), did not present hyperoxaluria (32 ± 18 mg/24 h) (Table 2). However, the hyperoxaluria that causes this type of stone may be intermittent and may not have been detected during the metabolic study. Thus, it would have been interesting to study the timing of Ca and oxalate intake since it is well known that Ca is a chelator, and its intake on an empty stomach or with meals can modify the absorption of other nutrients, such as oxalate, as well as influence the intestinal state regarding colonization by the bacterium Oxalobacter formigenes [14].

### 4.3. COD Stones

The COD stone group showed significantly higher dairy product intake than the control group, and MA showed that this could be a risk factor for COD stones. Dairy products are believed to be calcium-rich foods. However, despite all groups ingesting more dairy products than the control group, this fact was not reflected in calcium intake. All groups consumed less Ca than the control group. So, this risk association to dairy products could be justified by the presence of other nutrients such as fats, salt, and added sugars that are present in some dairy products such as cured cheese and flavored or sweetened yogurts. Moreover, in the questionnaire used, dairy products included, apart from milk, yogurt and cheese, other products such as bechamel, cream, ice cream, and other desserts. So, these products may pose a risk for kidney stone formation because of the presence of other nutrients. Given these results, it would have been interesting to subdivide dairy products according to different macro- and micronutrient contents.

Furthermore, it should be considered that dairy product consumption could entail a punctual increase of vitamin D serum levels (which increases calcium absorption), and its consumption usually occurs outside of meals, such as breakfast, where products ingested are not rich in oxalate, which would decrease calciuria by forming insoluble products with calcium. These circumstances may imply specific peaks in calcium absorption during breakfast or snacks, although they are not reflected in a higher overall consumption of vitamin D.

One of the urinary factors associated with this type of stone is hypercalciuria. The highest calciuria in 24 h in this study was presented by the COD group (248 ± 111 mg/24 h), and the difference was significant when compared with the non-papillary COM group. In addition, the most concentrated calciuria in urine at two hours was shown by this group (18 ± 16 mg/dL) (Table 2).

This study has some limitations. The main limitation is that this cross-sectional study includes a small sample (n = 90), which precludes conclusions regarding the temporal nature of our findings, and no solid conclusions can be established. Furthermore, numerous dietary components have been linked to stone formation. However, the scientific evidence for most of them is debatable, and the results claimed are frequently considered with individual nutrients and not in the context of a diet along with other factors and nutrients. The absence of strong evidence in this area makes it difficult to understand the role that food may play in stone disease, which may postpone the development of potentially effective treatments for this illness.

Even so, we found that diets rich in meat and rich in dairy products are associated to the formation of COM and COD calculi, respectively. For these reasons, prospective studies are needed to establish the time sequence in the relationship between them and clinically relevant findings.

## 5. Conclusions

Interesting differences in diet were observed between the different calcium oxalate stone groups and the control group. Nevertheless, further studies must be developed to clearly elucidate the role of meat and dairy products in COM and COD calculi.

## Figures and Tables

**Table 1:** Baseline characteristics.

	Control (n = 50)		COM Papillar (n = 13)		COM Non-Papillar (n = 27)		COD (n = 50)	
	Mean	(n/N)/SD	Mean	(n/N)/SD	Mean	(n/N)/SD	Mean	(n/N)/SD
Male% (n/N)	62.0%	(31/50)	76.9%	(10/13)	20	74.1%	66.0%	(33/50)
Female% (n/N)	38.0%	(19/50)	23.1%	(3/13)	7	25.9%	34.0%	(17/50)
Age (years)	51 ± 17		53 ± 12		59.3 ± 13.1		50 ± 12	
BMI (kg/cm^2^)	24.3 ± 4.3		28.7 ± 3.4 *		27.7 ± 6.2		26.8 ± 5.1	
Charslon Index	1.8 ± 1.9		1.3 ± 1.4		2.5 ± 2.1 ^a^		1.2 ± 1.3	
DM% (n/N)	14.3%	(7/49)	15.4%	(2/13)	5	18.5%	4.0%	(2/50)
HT% (n/N)	24.5%	(12/49)	23.1%	(3/13)	10	37.0%	36.0%	(18/50)
CRD% (n/N)	4.1%	(2/49)	7.7%	(1/13)	2	7.4%	4.0%	(2/50)
Family history% (n/N)	7.5%	(3/40)	72.7%	(8/11) *	5	25.0% ^a^	60.5%	(26/43) *
Personal history% (n/N)	0.0%	(0/47)	53.8%	(7/13) *	12	44.4%	65.3%	(32/49) *

* *p* < 0.05; ^a^: *p* < 0.05 vs. COM papillar.

**Table 2:** Comparison of 24 h urine between stone groups.

	COM Papillar (n = 13)	COM Non-Papillar (n = 27)	COD (n = 50)
	Mean ± SD	Mean ± SD	Mean ± SD
pH	5.6 ± 0.5	5.5 ± 0.6	5.8 ± 0.7
Ca 2 h (mg/dL)	13.5 ± 8.3	8.6 ± 5.7	18.0 ± 16.0 ^b^
Cr 2 h (mg/dL)	140 ± 63	103 ± 38	136 ± 70 ^b^
Cit 2 h (mg/dL)	578 ± 313	361 ± 161	410 ± 252
Urate 24 h (mg/dL)	274 ± 392	141 ± 213	204 ± 354
Urate 24 h (mg/24 h)	431 ± 358	453 ± 350	479 ± 298
Fosfate 24 h (mg/dL)	64 ± 25	41 ± 17	50 ± 24
Fosfate 24 h (mg/24 h)	910 ± 355	796 ± 365	917 ± 305
Ca 24 h (mg/dL)	12.9 ± 6.7	7.3 ± 3.4	13.6 ± 7.6
Ca 24 h (mg/24 h)	195 ± 115	145 ± 68	248 ± 111 ^b^
Mg 24 h (mg/dL)	6.3 ± 2.6	4.5 ± 2.2	5.8 ± 3.3
Mg 24 h (mg/24 h)	92 ± 43	93 ± 46	105 ± 48
Cr 24 h (mg/dL)	113 ± 43	71 ± 25 ^a^	80 ± 35
Cr 24 h (mg/24 h)	1620 ± 592	1387 ± 601	1457 ± 407
Cit 24 h (mg/dL)	485 ± 230	280 ± 151 ^a^	341 ± 354
Cit 24 h (mg/24 h)	663 ± 299	574 ± 426	625 ± 598
Ox 24 h (mg/dL)	16.2 ± 6.2	16.1 ± 7.3	16.5 ± 8.0
Ox 24 h (mg/24 h)	25.5 ± 13.8	31.9 ± 18.0	31.4 ± 14.2
Diuresis 24 h (mL)	1546 ± 551	2070 ± 971	2012 ± 630
Ca/cit 2 h	0.026 ± 0.025	0.033 ± 0.034	0.070 ± 0.075
Ca/cit 24 h	0.031 ± 0.020	0.035 ± 0.033	0.076 ± 0.113 ^b^

Ca: calcium; Cit: citrate; Mg: magnesium; Cr: creatinine; Ox: oxalate; Ca/cit: ratio calcium/citrate; ^a^: *p* < 0.05 vs. COM papillar; ^b^: *p* < 0.05 vs. COM non-papillar.

**Table 3:** Dietary factors associated with papillary COM stones (vs. control group).

	OR Adjusted	(95% IC of OR)	*p*-Value
Carbohydrates (g/d)	0.996	(0.987–1.006)	0.459
Protein (g/d)	1.015	(0.982–1.049)	0.386
Lipids (g/d)	1.014	(0.987–1.041)	0.309
Monounsaturated fatty acids (g/d)	1.012	(0.972–1.053)	0.562
Polyunsaturated fatty acids (g/d)	0.984	(0.867–1.117)	0.803
Saturated fatty acids (g/d)	1.060	(0.992–1.132)	0.085
Zinc (mg/d)	1.044	(0.793–1.375)	0.760
Niacin (mg/d)	0.997	(0.934–1.063)	0.920
Cholesterol (mg/d)	1.003	(0.999–1.006)	0.131
Trans fatty acids (g/d)	9.732	(2.071–45.732)	0.004 *
Legumes (g/d)	1.003	(0.970–1.037)	0.876
Meat and derivatives (g/d)	1.012	(1.003–1.021)	0.012 *
Sausages (g/d)	1.051	(1.004–1.099)	0.032 *
Glycemic load	0.994	(0.977–1.012)	0.539
Omega 3 fatty acids (g/d)	1.483	(0.931–2.361)	0.097
Caffeine and theine (mg/d)	1.009	(0.994–1.025)	0.235

All *p* < 0.1 dietary variables in the univariate analysis were considered. Values are shown as odds ratio (OR) adjusted for age, sex, BMI, and total energy (kcal/day), and their confidence interval is 95% (95% CI). * *p* < 0.05.

**Table 4:** Dietary factors associated with non-papillary COM stones (vs. control group).

	OR Adjusted	(95% CI of OR)	*p*-Value
Calcium (mg/d)	0.997	(0.994–0.999)	0.002 *
Iodine (mcg/d)	0.995	(0.991–0.999)	0.011 *
Legumes (g/d)	1.012	(0.987–1.036)	0.355
Dairy products (g/d)	1.002	(0.999–1.005)	0.192
Glycemic load	1.012	(0.998–1.026)	0.095
Caffeine and theine (mg/d)	1.013	(1.000–1.026)	0.044 *

All *p* < 0.1 dietary variables in the univariate analysis were considered. Values are shown as odds ratio (OR) adjusted for age, sex, BMI, and total energy (kcal/day), and their confidence interval is 95% (95% CI). * *p* < 0.05.

**Table 5:** Dietary factors associated with COD stones (vs. control group).

	OR Adjusted	(95% CI of OR)	*p*-Value
Legumes (g/d)	1.022	(0.999–1.046)	0.067
Dairy products (g/d)	1.005	(1.002–1.008)	<0.001 *
Yogurt (g/d)	0.995	(0.989–1.002)	0.145
Linoleic acid (g/d)	1.067	(0.985–1.157)	0.112
Glycemic load	1.004	(0.992–1.015)	0.536
Betacarotene (mcg/d)	0.999	(1.000–1.000)	0.037 *
Fytate (mg/d)	1.000	(1.000–1.001)	0.203

All *p* < 0.1 dietary variables in the univariate analysis were considered. Values are shown as odds ratio (OR) adjusted for age, sex, BMI, and total energy (kcal/day), and their confidence interval is 95% (95% CI). * *p* < 0.05.

## Data Availability

The data that support the findings of this study are available from the corresponding author, [I.C.], upon reasonable request.

## References

[1] Prochaska M.L., Taylor E.N., Curhan G.C. (2016). Insights Into Nephrolithiasis From the Nurses’ Health Studies. Am. J. Public Health.

[2] Yoshida O., Terai A., Ohkawa T., Okada Y. (1999). National trend of the incidence of urolithiasis in Japan from 1965 to 1995. Kidney Int..

[3] Grases F., Costa-Bauzá A., Ramis M., Montesinos V., Conte A. (2002). Simple classification of renal calculi closely related to their micromorphology and etiology. Clin. Chim. Acta.

[4] Costa-Bauzá A., Grases F., Julià F. (2023). The power of desktop scanning electron microscopy with elemental analysis for analyzing urinary stones. Urolitihiasis.

[5] Trinchieri A., Mandressi A., Luongo P., Longo G., Pisani E. (1991). The influence of diet on urinary risk factors for stones in healthy subjects and idiopathic renal calcium stone formers. Br. J. Urol..

[6] Trinchieri A., Nespoli R., Ostini F., Rovera F., Zanetti G., Pisani E. (1998). A study of dietary calcium and other nutrients in idiopathic renal calcium stone formers with low bone mineral content. J. Urol..

[7] Curhan G.C., Willett W.C., Knight E.L., Stampfer M.J. (2004). Dietary factors and the risk of incident kidney stones in younger women: Nurses’ Health Study II. Arch. Intern. Med..

[8] Taylor E.N., Stampfer M.J., Curhan G.C. (2004). Dietary factors and the risk of incident kidney stones in men: New insights after 14 years of follow-up. J. Am. Soc. Nephrol..

[9] Damasio P.C., Amaro C.R., Cunha N.B., Pichutte A., Goldberg J., Padovani C. (2011). The role of salt abuse on risk for hypercalciuria. Nutr. J..

[10] Fernández J.D., Piñol J.L., Zazpe I., Corella D., Carrasco P., Toledo E. (2010). Relative validity of a semi-quantitative food-frequency questionnaire in an elderly Mediterranean population of Spain. Br. J. Nutr..

[11] Lin B.-B., Lin M.-E., Huang R.-H., Hong Y.-K., Lin B.-L., He X.-J. (2020). Dietary and lifestyle factors for primary prevention of nephrolithiasis: A systematic review and meta-analysis. BMC Nephrol..

[12] Littlejohns T.J., Neal N.L., Bradbury K.E., Heers H., Allen N.E., Turney B.W. (2020). Fluid Intake and Dietary Factors and the Risk of Incident Kidney Stones in UK Biobank: A Population-based Prospective Cohort Study. Eur. Urol. Focus.

[13] Gambaro G., Ferraro P.M., Capasso G. (2012). Calcium nephrolithiasis, metabolic syndrome and the cardiovascular risk. Nephrol. Dial. Transpl..

[14] Allison M.J., Cook H.M., Milne D.B., Gallagher S., Clayman R.V. (1986). Oxalate degradation by gastrointestinal bacteria from humans. J. Nutr..

